# Physiotherapy students’ acceptance of AI-based chatbots (including ChatGPT) in education: a multi-institutional study from Turkey

**DOI:** 10.1186/s12909-025-08535-3

**Published:** 2026-01-03

**Authors:** Mehmet Akif Güler, Sümeyra Dağ, Senem Kayal, Merve Can, Sena Nur Şimşekkaya

**Affiliations:** Department of Physiotherapy and Rehabilitation, Faculty of Health Sciences, Selçuk University, Konya, Turkey

**Keywords:** Artificial intelligence, ChatGPT, Digital learning tools, Educational technology, Higher education, Physiotherapy education, Technology acceptance model

## Abstract

**Background:**

AI-based chatbots are increasingly used in health professions education to support learning tasks (e.g., studying, drafting assignments, and summarizing content). Evidence on the acceptance of AI-based chatbots, including ChatGPT, in physiotherapy education is limited. This study examined physiotherapy students’ acceptance in Turkey, focusing on perceived usefulness (PU) and perceived ease of use (PEOU) within the Technology Acceptance Model (TAM).

**Methods:**

A cross-sectional online survey (Google Forms) using convenience sampling was conducted with undergraduate physiotherapy students from nine universities across different regions of Turkey (May–June 2025). Digital informed consent was obtained, and responses were anonymous. PU and PEOU were assessed using Turkish TAM items contextualized for AI-based chatbot use. Psychometric properties were re-evaluated in the present sample (Cronbach’s alpha; PCA with KMO/Bartlett, with an EFA sensitivity analysis using PAF with Promax rotation), and group differences, correlations, and predictors of PU and PEOU were analyzed.

**Results:**

A total of 478 students (79.3% female) were included. Mean PU and PEOU scores were 52.91 ± 7.47 and 51.73 ± 6.49, respectively (scale range 14–70). PU and PEOU were moderately correlated (*r* = 0.450, *p* < 0.001). Technology interest was the strongest predictor of PU (*β* = 0.283, *p* < 0.001) and PEOU (β = 0.371, *p* < 0.001), while year of study had a smaller effect on PU (*β* = 0.112, *p* = 0.011); gender was not significant in regression models. Exploratory factor-analytic results suggested PU was unidimensional, whereas PEOU showed two exploratory facets (learnability/clarity and control/flexibility).

**Conclusions:**

Physiotherapy students expressed cautious optimism toward AI-based chatbots such as ChatGPT. Acceptance was higher among students with greater technology interest and, to a lesser extent, in advanced study years. These findings support stage-sensitive integration and targeted AI literacy, emphasizing ethical awareness and source verification for safe and effective learning.

**Supplementary Information:**

The online version contains supplementary material available at 10.1186/s12909-025-08535-3.

## Background

Artificial intelligence (AI) has become one of the most critical drivers of digital transformation in higher education. By enabling personalized learning, offering interactive content, and providing students with real-time feedback, AI-based tools are increasingly used in academic activities [1, 2]. In particular, large language model (LLM)–based chatbots, such as ChatGPT, have emerged as a prominent source of support in students’ study routines, exam preparation, and assignment completion. The literature suggests that these tools may support learning-related processes (e.g., brainstorming, summarizing, drafting) and time management, while also raising concerns regarding information accuracy, surface-level learning, academic integrity, and data privacy [3].

Despite these developments, most research on AI-assisted learning has focused on engineering, computer science, and general education programs [4]. Evidence in health sciences education remains relatively limited, particularly in physiotherapy education, where clinical reasoning, psychomotor skill acquisition, patient communication, and evidence-based decision-making are essential components of training. Physiotherapy students are required to develop theoretical knowledge and professional competencies simultaneously, which may influence how AI tools are perceived and adopted [5]. Understanding students’ acceptance is therefore pedagogically relevant for informing how such tools might be integrated into curricula and learning activities [6].

The Technology Acceptance Model (TAM), developed by Davis [7], is among the most widely applied frameworks for explaining acceptance of new technologies. TAM posits that individuals’ beliefs about a technology—primarily perceived usefulness (PU) and perceived ease of use (PEOU)—shape acceptance-related outcomes [7, 8]. PU refers to the extent to which a person believes that using a system will enhance performance, whereas PEOU reflects the extent to which using the system is perceived as free of effort [7]. Although later versions and extensions of TAM often include behavioral intention as a proximal determinant of actual use, PU and PEOU remain the core belief constructs that explain why a technology is evaluated favorably or not [7, 8]. In the present study, we focused on PU and PEOU as foundational indicators of acceptance because our primary aim was to characterize students’ belief-based evaluations of AI-based chatbots for academic purposes in an early-phase, descriptive national survey. This focus also enabled a concise assessment while maintaining alignment with the study’s research questions.

Empirical research on physiotherapy students’ acceptance of AI tools remains in its early stages. For instance, El-Sobkey et al. [9] conducted a large-scale international study exploring physiotherapy students’ acceptance of AI-based chatbots. While multi-country studies provide valuable cross-context insights, acceptance of AI-based tools in education may also be shaped by context-specific factors such as language, local educational practices, institutional policies, and infrastructural access [10]. Prior work has highlighted that students’ awareness of institutional guidance and the availability of resources can vary substantially across settings, influencing both perceived benefits and ethical concerns [11, 12]. Therefore, national-level evidence remains important to support context-sensitive integration strategies and to inform curriculum planning and AI literacy efforts within a given educational system. Despite increasing use of LLM-based chatbots in health professions education, national multi-institutional TAM-based evidence in physiotherapy students remains scarce, particularly regarding PU/PEOU and their demographic correlates. To the best of our knowledge, no national-level study has yet examined physiotherapy students’ acceptance of AI-based chatbots within the TAM framework, underscoring the originality of the present research. Therefore, this study aimed to examine physiotherapy students’ acceptance of AI-based chatbots for academic purposes in Turkey by assessing PU and PEOU within the TAM framework and evaluating their associations with gender, year of study, and technology interest, as well as identifying predictors of PU and PEOU.

We selected gender, year of study, and technology interest as predictors based on prior evidence that acceptance of educational technologies may vary by learners’ demographic and experiential characteristics. Gender has been examined in technology adoption research as a potential proxy for differences in prior exposure, self-efficacy, and patterns of technology engagement, although reported effects are often small and context-dependent [13]. Year of study represents students’ stage of training and curriculum demands; as learners progress from foundational coursework to more complex, clinically oriented tasks, they may develop different expectations about how AI tools can support learning, reflection, and preparation [5]. Finally, technology interest reflects an individual’s curiosity and motivation to engage with digital tools and has been linked to greater exploration and perceived value of new technologies, particularly in contexts where formal AI training opportunities are limited [14]. Taken together, these predictors provide a pragmatic and literature-informed set of factors to examine variability in PU and PEOU among physiotherapy students.

Specifically, the study addressed the following research questions:

1. What are the students’ PU and PEOU levels?
2. Do PU and PEOU differ across demographic factors such as gender, year of study, and technology interest?
3. What is the relationship between PU and PEOU, and which individual factors significantly predict these constructs?

Based on TAM, we hypothesized that gender, year of study, and technology interest would be associated with variations in PU and PEOU scores. This study provides national multi-institutional evidence from Turkey on physiotherapy students’ acceptance of AI-based chatbots, with practical implications for their pedagogical integration in physiotherapy education.

## Methods

### Study design

This cross-sectional quantitative study examined the perceptions of undergraduate physiotherapy students in Turkey regarding AI-based chatbots for academic purposes, within the TAM framework. This cross-sectional design was selected as a feasible approach for an early-phase, national snapshot of acceptance-related beliefs (PU/PEOU) across multiple institutions.

### Ethics statement

The study was approved by the Non-Interventional Clinical Research Ethics Committee of the Faculty of Health Sciences at Selçuk University (Date: March 5, 2025; No: 2025/02; Decision No: 2025/237). Participation was voluntary, digital informed consent was obtained, and procedures complied with the Declaration of Helsinki.

### Clinical trial number

Not applicable.

### Participants and sampling

The target population included approximately 29,807 undergraduate physiotherapy students in Turkey in 2025 [15, 16]. Using Cochran’s formula [17] with 95% confidence and a 5% margin of error, the required sample was 380, which was increased by 20% for potential non-response, yielding 456. In total, 520 students responded; after excluding 42 who reported no academic chatbot use, 478 complete responses from nine universities were included. The contextualized Turkish TAM items and their English translation are provided in Supplementary File 1. Convenience sampling was used due to feasibility constraints. Participants were recruited from nine universities across different regions of Turkey, and the survey link was disseminated via institutional contacts and student networks to enhance geographic and institutional diversity. The participating universities and respondent distribution are reported in Table S1 (Supplementary File 2).

### Inclusion and exclusion criteria

Inclusion criteria were: being an undergraduate physiotherapy student in Turkey, age ≥ 18 years, self-reported academic use of AI-based chatbots, and provision of informed consent. Because academic use of AI-based chatbots was an inclusion criterion, the analytic sample was restricted to users by design. Therefore, “chatbot use (user vs. non-user)” could not be modeled as an independent variable in the regression analyses, as there was no variability in chatbot use status within the final sample. Respondents who reported no academic chatbot use were excluded (42/520), yielding a final analytic sample of users (*n* = 478). Technology interest was included as a separate motivational predictor of PU/PEOU, rather than as a proxy for chatbot use. Exclusion criteria were enrollment in non-physiotherapy programs, failure to meet eligibility criteria (e.g., no academic chatbot use), refusal of consent, or responses flagged as inconsistent during data-quality checks.

### Data collection

Data were collected online (Google Forms) between May–June 2025. The survey included: (1) demographic information (gender, year of study, technology interest), (2) perceived usefulness (PU; 14 items), and (3) perceived ease of use (PEOU; 14 items). The survey was based on the Turkish adaptation of TAM by Parlak [18] and was contextualized for AI-based chatbot use in this study; reliability and construct validity were re-evaluated in the present sample.

Three experts (two physiotherapists with experience in physiotherapy education and one educational sciences scholar) independently reviewed the contextualized items for content relevance and clarity in relation to typical academic uses of AI-based chatbots in physiotherapy programs. Minor wording refinements were made based on consensus feedback, without altering item meaning, response format, or the original subscale structure. Specifically, generic references to “the system/technology” were reworded to “AI-based chatbots (including ChatGPT) used for academic purposes,” and performance-related wording was adapted to reflect students’ learning tasks (e.g., studying, assignments, and coursework). No new items were added; only contextual wording adjustments were made while retaining the original item structure and response format.

Items were rated on a five-point Likert scale (1 = strongly disagree, 5 = strongly agree). Negatively worded PEOU items were reverse-coded. Subscale scores ranged from 14 to 70, with higher scores indicating greater PU or PEOU. No formal pilot testing was conducted prior to data collection; however, the online form was checked to ensure proper functioning and item completeness.

### Variables

Primary outcome variables were perceived usefulness (PU) and perceived ease of use (PEOU). PU and PEOU were operationalized as total subscale scores by summing the 14 items in each subscale (possible range: 14–70), with higher scores indicating higher perceived usefulness or ease of use; negatively worded PEOU items were reverse-coded before score computation. The main predictors were gender (coded as 1 = male, 2 = female), year of study (1st–4th year), and self-reported technology interest (1 = very low to 5 = very high). The two PEOU components identified in PCA (learnability/clarity and control/flexibility) were treated as exploratory and were not used as separate dependent variables in the primary analyses.

### Bias

Selection bias may have arisen from convenience sampling across nine universities. Response bias is possible given the self-reported design; however, anonymity, voluntariness, and mandatory-response settings minimized missing data. As data were collected via a single self-report questionnaire, we acknowledge the potential for common-method bias; we minimized this risk procedurally through anonymity and voluntary participation. Further details on the institutional distribution of the sample are provided in Table S1 (Supplementary File 2).

### Statistical analysis

Descriptive statistics included frequencies (%) and means ± standard deviation (SD). Construct validity was tested using principal components analysis (PCA) with Varimax rotation, with the Kaiser–Meyer–Olkin (KMO) measure and Bartlett’s test of sphericity to assess sampling adequacy; loadings < 0.40 are omitted from Table 3 for readability. As a sensitivity check using a common-factor approach, we additionally performed an EFA using Principal Axis Factoring (PAF) with Promax rotation (oblique); full outputs are provided in Supplementary File 3 (Table S3). Score distributions were examined using Shapiro–Wilk tests and by inspecting skewness and kurtosis. Given the large sample size, Shapiro–Wilk tests were expectedly sensitive to minor departures from normality; therefore, distributional shape and model diagnostics were emphasized in decision-making. For between-group comparisons, non-parametric tests were used because PU/PEOU scores showed non-normal distributions and because key grouping variables were ordinal and/or had unequal group sizes (gender: Mann–Whitney *U*; year of study and technology interest: Kruskal–Wallis with Bonferroni-adjusted post hoc tests). For association and prediction analyses, PU and PEOU total scores were treated as continuous outcomes; Pearson correlations and multiple linear regression were applied, and assumptions were evaluated using residual diagnostics (linearity, homoscedasticity, approximate normality of residuals, and multicollinearity via VIF/tolerance). Although TAM is a well-established framework, the items in this study were contextualized for AI-based chatbot use and the dimensionality of PEOU in this specific context was not assumed a priori; therefore, PCA was used as an exploratory step to examine the underlying item structure and provide preliminary construct validity evidence. Confirmatory approaches (confirmatory factor analysis [CFA] or structural equation modeling [SEM]) were beyond the scope of this early-phase descriptive survey and are recommended for future studies to formally test model fit and measurement invariance. Internal consistency was assessed with Cronbach’s alpha. Because the online form used mandatory-response settings, there were no missing item-level data in the final dataset. We screened responses for eligibility and internal consistency; records failing eligibility criteria (e.g., non-user status) or flagged as inconsistent during data-quality checks were excluded prior to analysis. Analyses were performed in IBM SPSS Statistics (version 29.0) with *α* = 0.05.

## Results

**Fig. 1:**
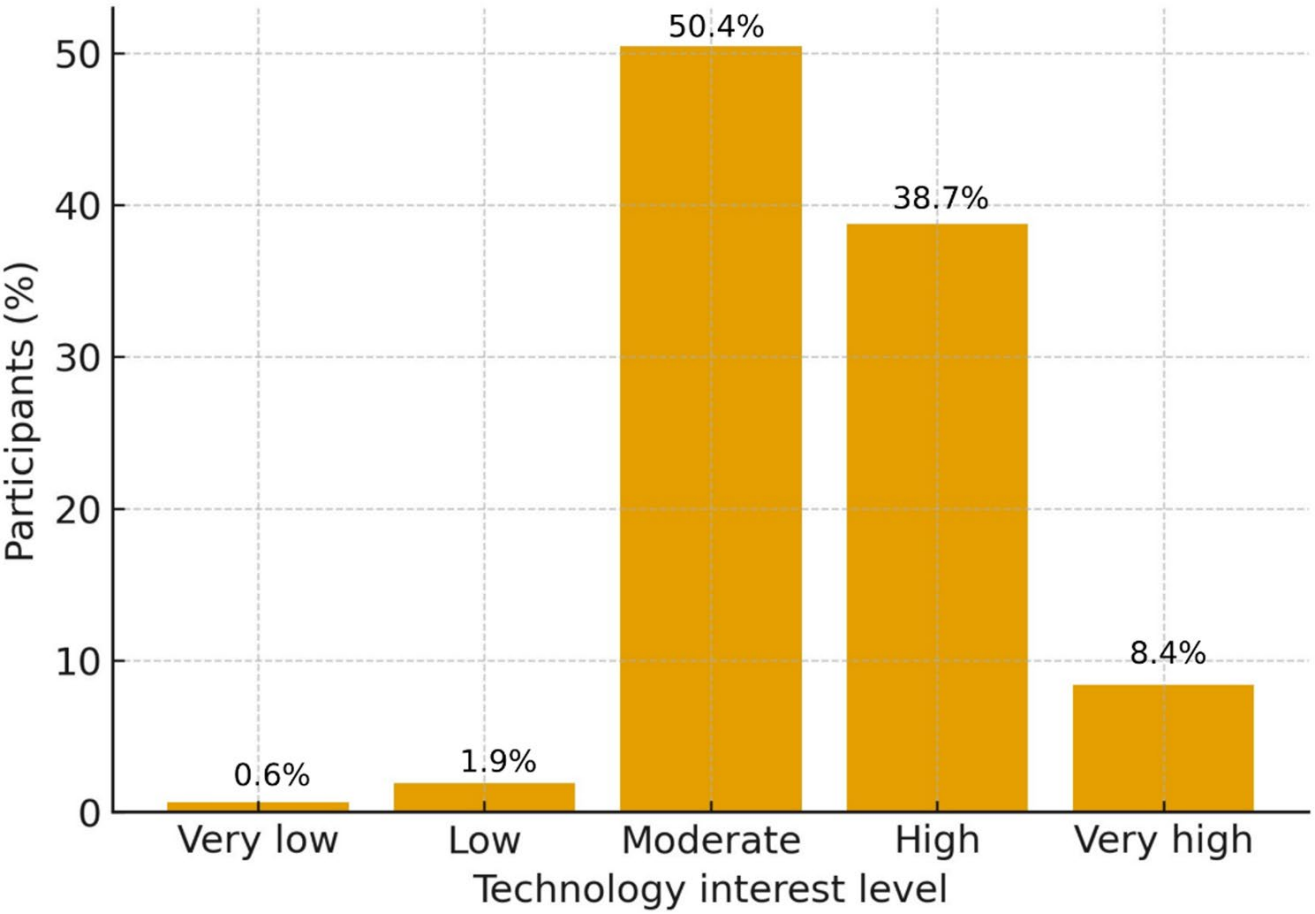
Distribution of self-reported technology interest among physiotherapy students (*n* = 478). Note. Most participants reported moderate (50.4%) or high (38.7%) technology interest, while very low (0.6%) and low (1.9%) levels were uncommon

A total of 478 undergraduate physiotherapy students were included in the final analysis. Most participants were female (79.3%), and the largest subgroup was second-year students (37.0%). Technology interest was predominantly moderate (50.4%) or high (38.7%), with very low and low levels reported by only 2.5% of the sample (Fig. 1).

PU scores ranged from 25 to 70 (*M* = 52.91, *SD* = 7.47), and PEOU scores ranged from 31 to 70 (*M* = 51.73, *SD* = 6.49). The 95% confidence intervals (CIs) were 52.24–53.58 for PU and 51.15–52.31 for PEOU. No missing data were observed for demographics or scale items. Detailed demographic distributions and descriptive statistics are provided in Table 1.

**Table 1:** Participant demographics, technology interest, and descriptive statistics of PU and PEOU scores

Variable	*n* (%)	Mean ± SD
Gender		
Female	379 (79.3)	
Male	99 (20.7)	
Year of Study		
1st year	132 (27.6)	
2nd year	177 (37.0)	
3rd year	104 (21.8)	
4th year	65 (13.6)	
Technology Interest		
Very low	3 (0.6)	
Low	9 (1.9)	
Moderate	241 (50.4)	
High	185 (38.7)	
Very high	40 (8.4)	
Scale Scores		
PU		52.91 ± 7.47
PEOU		51.73 ± 6.49

*PU* Perceived Usefulness, *PEOU * Perceived Ease of Use, *SD* Standard deviation, *n* Number of participants

### Reliability and validity

Both subscales demonstrated strong internal consistency, with Cronbach’s *α* = 0.922 for PU and *α* = 0.856 for PEOU. Item–total correlations ranged from 0.322 to 0.760 for PU and from 0.306 to 0.638 for PEOU, indicating adequate homogeneity across items. Sampling adequacy was excellent (KMO = 0.927), and Bartlett’s test confirmed factorability (*χ²*(378) = 7161.82, *p* < 0.001).

Principal Components Analysis with Varimax rotation revealed a three-factor solution explaining 54.98% of the variance. Based on the Kaiser criterion (eigenvalues > 1), three components were retained (initial eigenvalues = 9.324, 3.825, and 2.244), and the scree plot indicated a clear elbow after the third component. Exploratory analyses yielded three factors overall: PU was unidimensional, while PEOU split into two exploratory facets (learnability/clarity and control/flexibility), with all loadings ≥ 0.40. Cross-loadings were not observed at the ≥ 0.40 threshold (i.e., no item loaded ≥ 0.40 on more than one component). Rotated variance explained by each factor is reported in Table 2.

As a sensitivity analysis, we additionally conducted an exploratory factor analysis using Principal Axis Factoring (PAF) with Promax rotation (oblique), allowing correlated factors. The PAF–Promax solution was consistent with the PCA findings, yielding the same three-factor structure (PU items on Factor 1; PEOU items clustered into two related factors) with loadings ≥ 0.40 and moderate factor intercorrelations (*r* = 0.317–0.529). Full PAF–Promax outputs (pattern matrix, communalities, factor correlations, eigenvalues, and variance explained) are provided in Supplementary File 3 (Table S3). Accordingly, the EFA (PAF–Promax) sensitivity analysis corroborated the three-factor structure observed in PCA.

Reliability coefficients and PCA-based variance explained are presented in Table 2, and detailed PCA factor loadings are provided in Table 3. For subsequent analyses, we operationalized PEOU using the total PEOU score, consistent with the original TAM conceptualization and the study’s research questions. The two PEOU components identified by PCA (learnability/clarity and control/flexibility) were treated as exploratory subdimensions and were not analyzed as separate dependent variables to preserve parsimony, maintain comparability with prior TAM-based research, and avoid inflating Type I error due to additional multiple testing. Confirmatory work is recommended to validate this PEOU substructure.

**Table 2:** Reliability coefficients and factor structure of PU and PEOU subscales

Factor/Subscale	Construct	Rotated variance explained (%)	k	α	ITC range
F1	PU	25.56	14	0.922	0.322–0.760
F2	PEOU – Learnability/Clarity	16.66	8	–	–
F3	PEOU – Control/Flexibility	12.76	6	–	–
Subscale	PEOU (total)	29.42	14	0.856	0.306–0.638
Total variance explained	**54.98**	–	–	–	

F1 = PU (Perceived Usefulness); F2 = PEOU (Learnability/Clarity); F3 = PEOU (Control/Flexibility). Rotated variance percentages are from the Rotation Sums of Squared Loadings (Varimax). Dashes (–) indicate that reliability coefficients were not calculated for the exploratory PEOU facets

*PU* Perceived Usefulness, *PEOU* Perceived Ease of Use, *α* Cronbach’s alpha, *k* number of items, *ITC* item–total correlation, *PCA * Principal Components Analysis

**Table 3:** Rotated component matrix (PCA, Varimax) for PU and PEOU items

Item	F1	F2	F3	Item	F1	F2	F3
PU1	0.419	—	—	PU8	0.729	—	—
PU2	0.687	—	—	PU9	0.546	—	—
PU3	0.732	—	—	PU10	0.798	—	—
PU4	0.683	—	—	PU11	0.792	—	—
PU5	0.569	—	—	PU12	0.802	—	—
PU6	0.751	—	—	PU13	0.726	—	—
PU7	0.727	—	—	PU14	0.624	—	—
PEOU1	—	0.745	—	PEOU8	—	—	0.710
PEOU2	—	0.797	—	PEOU9	—	0.680	—
PEOU3	—	0.756	—	PEOU10	—	0.780	—
PEOU4	—	0.792	—	PEOU11	—	—	0.769
PEOU5	—	0.733	—	PEOU12	—	—	0.654
PEOU6	—	—	0.577	PEOU13	—	—	0.745
PEOU7	—	0.664	—	PEOU14	—	—	0.745

F1 = PU items (Perceived Usefulness); F2 = PEOU items (Learnability/Clarity); F3 = PEOU items (Control/Flexibility)

*PU* Perceived Usefulness, *PEOU* Perceived Ease of Use; “—” = no loading ≥ 0.40. Loadings below 0.40 are omitted

### Group comparisons

Non-parametric tests were applied because of non-normal distributions and ordinal predictors. Gender differences emerged for PU, with male students scoring significantly higher than females, although the effect size was small. However, the magnitude of this gender difference was small (*r* = − 0.12) and did not remain significant in the multiple regression model, suggesting limited practical relevance. No gender differences were observed for PEOU.

**Fig. 2:**
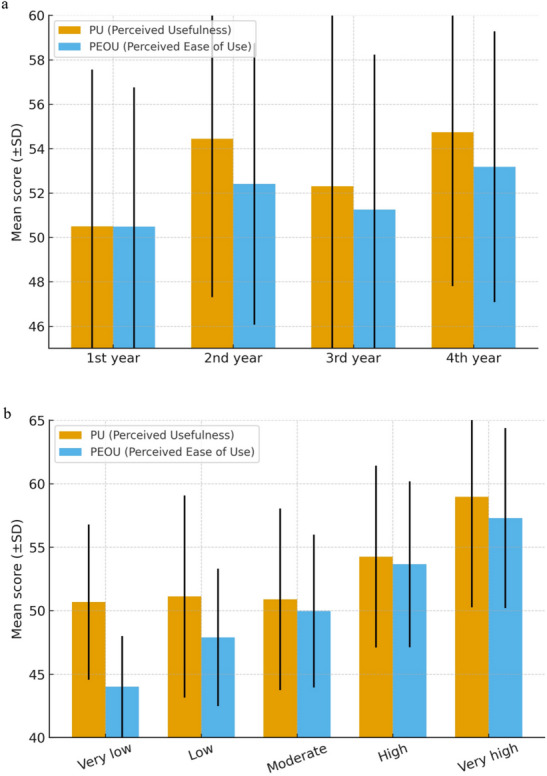
PU and PEOU scores by (**a**) year of study and (**b**) technology interest level. Note. **a** First-year students reported lower PU and PEOU compared with second- and fourth-year students, consistent with Kruskal–Wallis and post hoc test results. **b** Students with high and very high technology interest scored significantly higher than those with moderate or lower levels, consistent with Kruskal–Wallis and post hoc test results. PU = Perceived Usefulness; PEOU = Perceived Ease of Use

Year of study significantly influenced both PU and PEOU. First-year students scored lower than their second- and fourth-year peers on PU, and also lower than these groups on PEOU, as illustrated in Fig. 2a.

Technology interest showed the strongest effects. Students with high or very high levels of interest scored substantially higher on both PU and PEOU compared with those reporting moderate or lower levels (Fig. 2b). These effects reached medium to large magnitude. Detailed statistics and effect sizes are presented in Table 4.

**Table 4:** Non-parametric group comparisons of PU and PEOU scores with effect sizes

Variable	Subscale	Test	Test statistic	Post hoc results	*p*	Effect size
Gender	PU	Mann–Whitney *U*	*U* = 15505.5; *Z =* − 2.67	–	0.008	*r =* − 0.12 (small)
	PEOU	Mann–Whitney *U*	*U* = 17133.5; *Z =* − 1.33	–	0.183	*r =* − 0.06 (negligible)
Year of study	PU	Kruskal–Wallis	*χ²*(3) = 25.71	1st < 2nd; 1st < 4th (both *p*_adj < 0.001)	< 0.001	*η²* = 0.054 (small–medium)
	PEOU	Kruskal–Wallis	*χ²*(3) = 11.16	1st < 2nd (*p*_adj = 0.025); 1st < 4th (*p*_adj = 0.037)	0.011	*η²* = 0.023 (small)
Technology interest	PU	Kruskal–Wallis	*χ²*(4) = 46.43	High/Very high > Moderate/Low	< 0.001	*η²* = 0.097 (medium)
	PEOU	Kruskal–Wallis	*χ²*(4) = 64.50	High/Very high > Moderate/Low	< 0.001	*η²* = 0.135 (medium–large)

*PU* Perceived Usefulness, *PEOU* Perceived Ease of Use, *U* Mann–Whitney *U* statistic, *Z* standardized test statistic, *χ²* chi-square statistic from Kruskal–Wallis test, *p* significance value for overall test, *p_adj* Bonferroni-adjusted significance value for post hoc comparisons, *r* effect size for Mann–Whitney *U*, *η²* effect size for Kruskal–Wallis

### Correlation analysis

PU and PEOU were moderately and positively correlated (*r* = 0.450, *p* < 0.001), indicating that students who perceived AI-based chatbots as more useful also found them easier to use. The association between PU and PEOU remained significant after controlling for technology interest (partial *r* = 0.382, *p* < 0.001). The correlation is presented in Table 5.

**Table 5:** Correlation between PU and PEOU scores

Variable	PU_Total	PEOU_Total
PU_Total	1	0.450**
PEOU_Total	0.450**	1

*r* values represent Pearson correlation coefficients

*PU_Total* Total score of Perceived Usefulness, *PEOU_Total* Total score of Perceived Ease of Use

**p < 0.001 (two-tailed)

### Regression analysis

Regression analyses showed that technology interest was the strongest predictor of both PU and PEOU. For PU, the overall model was significant, *F*(3,474) = 18.65, *p* < 0.001, explaining 10.6% of the variance (*R²* = 0.106; *f²* = 0.12, small-to-medium effect). Technology interest (β = 0.283, *p* < 0.001) and year of study (*β* = 0.112, *p* = 0.011) were significant predictors, whereas gender was not.

For PEOU, the model was also significant, *F*(3,474) = 25.21, *p* < 0.001, accounting for 13.8% of the variance (*R²* = 0.138; *f²* = 0.16, medium effect). In this model, only technology interest was a significant predictor (*β* = 0.371, *p* < 0.001), while gender and year of study were non-significant. No issues with multicollinearity were observed (all VIF < 1.2). Detailed coefficients are reported in Table 6.

**Table 6:** Multiple regression predicting PU and PEOU with effect sizes

Dependent variable	Predictor	B	SE	β	t	*p*	Model fit / Effect size
PU	Constant	41.657	2.649	–	15.727	< 0.001	*F*(3,474) = 18.65, *p* < 0.001; *R²* = 0.106; *f²* = 0.12 (small–medium)
	Gender	–0.663	0.838	–0.036	–0.792	0.429	
	Year of study	0.837	0.327	0.112	2.559	0.011	
	Technology interest	3.008	0.486	0.283	6.190	< 0.001	
PEOU	Constant	37.291	2.259	–	16.508	< 0.001	*F*(3,474) = 25.21, *p* < 0.001; *R²* = 0.138; *f²* = 0.16 (medium)
	Gender	0.812	0.714	0.051	1.136	0.257	
	Year of study	0.412	0.279	0.063	1.477	0.140	
	Technology interest	3.428	0.414	0.371	8.272	< 0.001	

Gender was coded as 1 = male and 2 = female. Year of study (1–4) and technology interest (1–5) were entered as ordinal-coded numeric predictors

*PU* Perceived Usefulness, *PEOU* Perceived Ease of Use, *B* Unstandardized coefficient, *SE* Standard error, *β * Standardized coefficient, *t* *t*-statistic, *F*(*df₁*,* df₂*) Model F-test, *R²* Coefficient of determination, *VIF* Variance Inflation Factor, *f²* effect size calculated as *R²* / (1 – *R²*)

## Discussion

This study evaluated the acceptance of AI-based chatbots for academic purposes among physiotherapy and rehabilitation students in Turkey within the framework of the TAM. Our findings indicate that students generally perceived AI-based chatbots as moderately to highly useful (PU) and relatively easy to use (PEOU). To improve clarity, the Discussion is organized around (i) interpretation of key findings, (ii) comparison with prior studies, and (iii) implications for physiotherapy education.

Lindbäck et al. [5], in their qualitative study, reported that students viewed AI as a “study partner” or “virtual friend,” yet demonstrated cautious optimism due to concerns about overreliance, accuracy limitations, and uncertainties regarding permitted use. The moderate–high PU and PEOU scores observed in our study can be interpreted as the quantitative reflection of these themes. With mean scores of 52.91 (*SD* = 7.47) for PU and 51.73 (*SD* = 6.49) for PEOU, physiotherapy students’ acceptance of AI chatbots appears to reflect this balanced optimism. This is particularly relevant for rehabilitation education, where students must simultaneously acquire theoretical knowledge and clinical reasoning skills. Structured integration of AI tools has the potential to enhance critical reflection in case-based learning and improve students’ preparedness for real-world rehabilitation practice.

Previous studies similarly highlight that while students consider generative AI tools beneficial for time management, idea generation, and learning motivation, they remain concerned about accuracy, surface-level learning, ethical dilemmas, and data privacy [1–4]. Our findings quantitatively support this dual evaluation. Given that our outcomes reflect students’ self-reported beliefs (PU/PEOU), we cannot infer effects on learning performance or clinical reasoning. However, concerns reported in the literature regarding accuracy, academic integrity, and privacy highlight the importance of emphasizing verification practices and responsible use when integrating AI chatbots into educational activities. This underscores the need for clear guidance on verification practices and responsible use in educational settings.

Gender differences were modest: male students reported higher PU scores than females, although the effect size was small (*r* = − 0.12). This aligns with Tortella et al. [13], who observed greater academic use of AI chatbots among male students in Italy. However, Lindbäck et al. [5] emphasized that such differences are more likely related to factors such as technology interaction, self-efficacy, and computer skills rather than gender per se. Accordingly, gender may act as a proxy, and targeted AI literacy initiatives could help mitigate these disparities.

Lower PU and PEOU scores among first-year students suggest that the value of AI tools becomes more evident as students progress through their training and encounter authentic tasks. Supporting this interpretation, Lindbäck et al. [5] noted that advanced students used AI as a “reflective tool” in clinical reasoning and case preparation, consistent with the year-level differences observed in our study.

Technology interest emerged as the strongest predictor of both PU (*β* = 0.283) and PEOU (*β* = 0.371), consistent with TAM’s central propositions [7, 8]. Similarly, Vera [14] demonstrated that in contexts with limited formal training opportunities, individual curiosity and motivation were key facilitators of technology adoption. Our results extend these findings by showing that technology interest not only enhances perceived ease of use but also contributes to perceived usefulness through the development of effective usage strategies. Although the explained variance was modest (*R²* = 0.106 for PU; *R²* = 0.138 for PEOU), this magnitude is typical in behavioural/educational survey research and suggests that additional factors (e.g., AI literacy, prior training, institutional guidance, and access) may also contribute to students’ acceptance.

We also found a moderate, positive, and significant correlation between PU and PEOU (*r* = 0.45), in line with Davis’s [7] core TAM assumption that technologies perceived as easier to use are also judged as more useful. Consistent with TAM, the moderate PU–PEOU association suggests that when students perceive AI chatbots as easier to use (lower effort), they also tend to evaluate them as more useful for academic tasks, supporting the core belief structure proposed by Davis.

Consistent with the Results, PU was unidimensional, whereas PEOU separated into two exploratory facets (learnability/clarity and control/flexibility). This diverges from the unidimensional assumption of TAM, suggesting that physiotherapy students value not only the ability to initiate AI interactions easily but also the capacity to guide these interactions according to discipline-specific objectives. However, given the exploratory nature of this finding, future research should verify this structure using confirmatory approaches. This pattern is partially consistent with TAM: PU emerged as a unidimensional construct, while PEOU showed a more differentiated structure in this AI-chatbot context. Conceptually, both PEOU subdimensions reflect facets of effort expectancy (ease of learning/understanding and perceived control over interaction); therefore, retaining a single composite PEOU score for the main analyses remains aligned with TAM’s core belief construct. Although PCA suggested two PEOU facets, we treated this as exploratory and retained the total PEOU score to remain consistent with TAM’s core construct; confirmatory testing (CFA/SEM) is required before considering these facets as distinct dimensions. Given the exploratory nature of this finding, it should be interpreted cautiously until confirmed with CFA/SEM.

Overall, our findings and the existing literature underscore the need for professional integration of chatbots into education rather than outright prohibition. From a competency-based perspective, AI tools may support clinical reasoning by offering rapid information retrieval and simulation of patient scenarios. However, without guided training, there is a risk of superficial learning and overreliance, which may compromise the development of essential rehabilitation competencies such as communication, critical thinking, and evidence-based practice. Four key recommendations emerge. First, integration should be tailored to the stage of training: early years could emphasize short prompt examples, ethical checklists, and verification exercises, whereas clinical years could incorporate AI-assisted SOAP (Subjective–Objective–Assessment–Plan) notes, evidence-based justification, and reflective writing tasks [5, 6]. Second, systematic development of AI literacy and prompting skills is essential. Structured and guided practice not only enhances PEOU but also strengthens PU [1–4], thereby reducing inequities linked to differing levels of technology interest. In addition, these findings may inform program-level quality assurance and accreditation processes by highlighting AI literacy, ethical use, and source verification as emerging digital competencies that can be embedded in physiotherapy curricula and assessed within educational standards. Third, ethical and safety dimensions require particular emphasis, including human selection of references, systematic accuracy checks, and strict avoidance of personal or health-related data input. Transparent institutional guidelines are also critical, as evidenced by a large-scale study in the UAE, where 79.6% of students reported using AI tools, yet 41.5% were unaware of or uncertain about institutional policies [11]. Finally, equitable access must not be overlooked. In Nigeria, awareness was reported at 46% and usage at 41%, with inadequate internet (61.9–62%) and cost (53–59.7%) identified as primary barriers [12]. From an educator perspective, Opesemowo et al. similarly reported that lecturers perceived ChatGPT as potentially useful for instructional assessment, provided that its use is aligned with clear pedagogical goals and ethical guidance [19]. Taken together, these findings suggest that in Turkey, addressing access to licensed tools, internet capacity, and device availability will be essential for equitable integration.

### Limitations

This study has several limitations. The use of convenience sampling introduces the possibility of selection bias. Its cross-sectional design precludes causal inferences between variables. Self-reported measures may be subject to common-method bias. Because all variables were collected via a single self-report survey at one time point, common-method bias may have inflated some associations among constructs. We did not conduct a formal statistical test for common-method bias (e.g., Harman’s single-factor test). However, we attempted to reduce this risk procedurally through anonymous and voluntary participation and clear item wording. Future studies should apply recommended statistical and design-based remedies (e.g., marker variables, temporal separation, or multi-source measures).

Because the sample included only students who reported academic use of AI-based chatbots, the findings may not generalize to non-users, and prevalence estimates of chatbot use should not be inferred from this dataset. Future studies should recruit both users and non-users and include usage-intensity indicators (e.g., frequency, duration, and specific academic tasks) to better evaluate acceptance across different levels of exposure.

Additionally, we could not fully account for potential institutional or regional differences (e.g., local teaching practices or informal policies) that may shape students’ AI use and perceptions. Furthermore, psychometric evaluation and hypothesis testing were conducted within the same analytic dataset, which may limit the robustness of the factor-analytic findings and the generalizability of the predictive models. Future studies should replicate the measurement structure and predictors in independent samples (e.g., split-sample EFA/CFA or external multi-institutional validation) to strengthen the evidence. Moreover, the two-dimensional structure within PEOU was identified through an exploratory approach and therefore requires confirmation in future research using CFA or SEM, including measurement invariance testing. Accordingly, this factor pattern—particularly the two-dimensional structure within PEOU—should be interpreted cautiously. Finally, findings are context-specific to Turkey and may have limited generalizability to countries with different cultural or technological infrastructures.

### Future research

Future studies should test the two-factor structure of PEOU through CFA or SEM and examine measurement invariance across gender, year of study, and institutional differences. Longitudinal designs are needed to track changes in technology acceptance throughout the course of physiotherapy education, particularly during the transition from preclinical to clinical years. Experimental research could evaluate the effects of AI literacy and prompt training interventions on PU, PEOU, accuracy, and ethical conduct. Additionally, task- and output-focused studies should explore how AI acceptance relates to academic performance indicators such as literature review, case report preparation, clinical reasoning, and patient education. Policy-oriented research could also investigate how infrastructural investments—such as licensing, bandwidth, and device support—impact technology acceptance and equity. Future research should also include cross-national comparisons and experimental evaluations of structured chatbot-based learning modules to test causal effects on learning outcomes and safe-use behaviours.

## Conclusions

Physiotherapy students in Turkey demonstrated moderate-to-high acceptance of AI-based chatbots for academic purposes. Technology interest was the strongest predictor of both PU and PEOU, while year of study also contributed to higher PU scores. These findings provide one of the first national-level insights into physiotherapy students’ AI acceptance and support the theme of “cautious optimism.” In educational settings, these results suggest that integrating AI chatbots may benefit from stage-sensitive teaching strategies, prompt training, and guidance that emphasizes ethical awareness and verification practices. Given the cross-sectional, self-report design and the Turkey-specific sample, the findings should be interpreted as context-bound and not generalized beyond similar settings without further research. These findings also align with global physiotherapy education reform and digital competency frameworks that emphasize responsible AI literacy and safe use in health professions education.

## Supplementary Information

Supplementary Material 1.

Supplementary Material 2.

Supplementary Material 3.

## Data Availability

The datasets generated and/or analyzed during the current study are available from the corresponding author on reasonable request.

## Abbreviations

AI: Artificial intelligence
CFA: Confirmatory Factor Analysis
CI: Confidence interval
EFA: Exploratory Factor Analysis
KMO: Kaiser–Meyer–Olkin
LLM: Large language model
PAF: Principal Axis Factoring
PCA: Principal Components Analysis
PEOU: Perceived ease of use
PU: Perceived usefulness
SD: Standard deviation
SEM: Structural Equation Modeling
SOAP: Subjective–Objective–Assessment–Plan
TAM: Technology Acceptance Model
VIF: Variance Inflation Factor
