# Supplementary material for: Physiotherapy students’ acceptance of AI-based chatbots (including ChatGPT) in education: a multi-institutional study from Turkey
Source: BMC Med Educ. 2026 Jan 3;26:174. doi: 10.1186/s12909-025-08535-3 (PMC12866455; doi:10.1186/s12909-025-08535-3)
Supplement: Supplementary file 1 — Supplementary Material 1. [file 12909_2025_8535_MOESM1_ESM.docx]

**Perceived Usefulness (PU) Subscale**

Below are statements regarding the use of **Artificial Intelligence–Based Learning Tools (AIBLTs)**.
Please indicate the extent to which you agree with each statement by selecting the appropriate option.

**1) Strongly Disagree**

**2) Disagree**

**3) Undecided**

**4) Agree**

**5) Strongly Agree**

| **#** | **Statement** |
| --- | --- |
| 1 | It would be difficult for me to manage my learning process without using AIBLTs. |
| 2 | Using AIBLTs helps me learn my courses more effectively. |
| 3 | Using AIBLTs improves my academic performance. |
| 4 | AIBLTs meet my educational needs. |
| 5 | Using AIBLTs saves me time in my learning process. |
| 6 | AIBLTs help me learn topics more quickly. |
| 7 | AIBLTs support the critical aspects of my learning process. |
| 8 | Using AIBLTs enables me to learn more topics and materials. |
| 9 | Using AIBLTs reduces unnecessary time loss. |
| 10 | Using AIBLTs increases my academic efficiency. |
| 11 | Using AIBLTs improves the quality of what I learn. |
| 12 | Using AIBLTs enhances my learning productivity. |
| 13 | Using AIBLTs makes it easier for me to understand course materials. |
| 14 | Overall, I find AIBLTs useful. |

**Perceived Ease of Use (PEOU) Subscale**

Below are statements regarding the use of **Artificial Intelligence–Based Learning Tools (AIBLTs)**.
Please indicate the extent to which you agree with each statement by selecting the appropriate option.

**1) Strongly Disagree**
**2) Disagree**
**3) Undecided**
**4) Agree**
**5) Strongly Agree**

| **#** | **Statement** |
| --- | --- |
| 1 | I often get confused when using AIBLTs. |
| 2 | I often make mistakes when using AIBLTs. |
| 3 | Using AIBLTs is often frustrating. |
| 4 | I frequently need to refer to a user manual when using AIBLTs. |
| 5 | Using AIBLTs requires too much mental effort. |
| 6 | It is easy for me to correct the mistakes I make when using AIBLTs. |
| 7 | AIBLTs are not flexible in use; they are rigid. |
| 8 | It is easy for me to use AIBLTs to access the learning materials I need. |
| 9 | AIBLTs often operate in unexpected ways. |
| 10 | I find AIBLTs impractical to use. |
| 11 | I find it easy to use AIBLTs. |
| 12 | It is easy for me to remember the steps when using AIBLTs. |
| 13 | AIBLTs provide helpful guidance in my learning process. |
| 14 | Overall, I find AIBLTs easy to use. |

**Note:** Items 1, 2, 3, 4, 5, 7, 9, and 10 in the *Perceived Ease of Use (PEOU)* subscale of the *Technology Acceptance Model (TAM)* are negatively worded and should be reverse-coded during analysis.
