# Supplementary material for: Physiotherapy students’ acceptance of AI-based chatbots (including ChatGPT) in education: a multi-institutional study from Turkey
Source: BMC Med Educ. 2026 Jan 3;26:174. doi: 10.1186/s12909-025-08535-3 (PMC12866455; doi:10.1186/s12909-025-08535-3)
Supplement: Supplementary file 2 — Supplementary Material 2. [file 12909_2025_8535_MOESM2_ESM.docx]

**Table S1.** Participating universities and sample distribution (*n* = 478)

| **University** | **City** | **Region (Turkey)** | ***n*** | **%** |
| --- | --- | --- | --- | --- |
| Selçuk University | Konya | Central Anatolia | 187 | 39.1 |
| KTO Karatay University | Konya | Central Anatolia | 51 | 10.7 |
| Bolu Abant İzzet Baysal University | Bolu | Black Sea | 24 | 5.0 |
| Sivas Cumhuriyet University | Sivas | Central Anatolia | 14 | 2.9 |
| Necmettin Erbakan University | Konya | Central Anatolia | 80 | 16.7 |
| Burdur Mehmet Akif Ersoy University | Burdur | Mediterranean | 19 | 4.0 |
| Ankara Yıldırım Beyazıt University | Ankara | Central Anatolia | 81 | 16.9 |
| Kayseri University | Kayseri | Central Anatolia | 5 | 1.0 |
| Kütahya Health Sciences University | Kütahya | Aegean | 17 | 3.6 |

**Note.** Values are *n* (%) of the final analytic sample.
