# Supplementary material for: Physiotherapy students’ acceptance of AI-based chatbots (including ChatGPT) in education: a multi-institutional study from Turkey
Source: BMC Med Educ. 2026 Jan 3;26:174. doi: 10.1186/s12909-025-08535-3 (PMC12866455; doi:10.1186/s12909-025-08535-3)
Supplement: Supplementary file 3 — Supplementary Material 3. [file 12909_2025_8535_MOESM3_ESM.docx]

**Table S3.** Exploratory factor analysis (EFA) sensitivity analysis using Principal Axis Factoring (PAF) with Promax rotation (*n* = 478).

**Panel A.** Pattern matrix (Promax) and communalalities (*h²*)

| **Item** | ***h²*** | **Factor 1 (PU)** | **Factor 2 (PEOU–L/C)** | **Factor 3 (PEOU–C/F)** |
| --- | --- | --- | --- | --- |
| PU1 | 0.137 | 0.413 |  |  |
| PU2 | 0.480 | 0.668 |  |  |
| PU3 | 0.578 | 0.711 |  |  |
| PU4 | 0.458 | 0.665 |  |  |
| PU5 | 0.403 | 0.508 |  |  |
| PU6 | 0.572 | 0.755 |  |  |
| PU7 | 0.498 | 0.736 |  |  |
| PU8 | 0.559 | 0.713 |  |  |
| PU9 | 0.330 | 0.493 |  |  |
| PU10 | 0.632 | 0.832 |  |  |
| PU11 | 0.603 | 0.840 |  |  |
| PU12 | 0.644 | 0.836 |  |  |
| PU13 | 0.531 | 0.721 |  |  |
| PU14 | 0.496 | 0.560 |  |  |
| PEOU1 | 0.511 |  | 0.726 |  |
| PEOU2 | 0.618 |  | 0.797 |  |
| PEOU3 | 0.566 |  | 0.748 |  |
| PEOU4 | 0.605 |  | 0.787 |  |
| PEOU5 | 0.473 |  | 0.712 |  |
| PEOU6 | 0.237 |  |  | 0.494 |
| PEOU7 | 0.373 |  | 0.620 |  |
| PEOU8 | 0.380 |  |  | 0.658 |
| PEOU9 | 0.351 |  | 0.610 |  |
| PEOU10 | 0.447 |  | 0.628 |  |
| PEOU11 | 0.680 |  |  | 0.858 |
| PEOU12 | 0.646 |  |  | 0.796 |
| PEOU13 | 0.580 |  |  | 0.601 |
| PEOU14 | 0.589 |  |  | 0.747 |

**Panel B.** Factor correlation matrix (Phi)

|  | **Factor 1** | **Factor 2** | **Factor 3** |
| --- | --- | --- | --- |
| Factor 1 | 1.000 | 0.317 | 0.529 |
| Factor 2 | 0.317 | 1.000 | 0.364 |
| Factor 3 | 0.529 | 0.364 | 1.000 |

**Panel C.** Total variance explained (PAF extraction)

| **Factor** | **Extraction SS Loadings** | **% of Variance** | **Cumulative %** |
| --- | --- | --- | --- |
| 1 | 8.857 | 31.632 | 31.632 |
| 2 | 3.338 | 11.922 | 43.554 |
| 3 | 1.782 | 6.364 | 49.918 |

**Note.** Loadings < 0.40 are suppressed. *h²* indicates communality (extraction). Extraction method: Principal Axis Factoring (PAF). Rotation: Promax with Kaiser normalization. Factor labels: Factor 1 = Perceived Usefulness (PU); Factor 2 = Perceived Ease of Use (PEOU) – Learnability/Clarity; Factor 3 = Perceived Ease of Use (PEOU) – Control/Flexibility. Because Promax is an oblique rotation, rotated sums of squared loadings are not additive; therefore, variance percentages are reported for the extraction solution.
